# DNA Binding of Some Chiral Metallointercalators Derived From 9,10-Phenanthrenediamine

**DOI:** 10.1155/MBD.1998.225

**Published:** 1998

**Authors:** Susan F. Murphy-Poulton, Robert S. Vagg, Kymberley A. Vickery, Peter A. Williams

**Affiliations:** 1 School of Chemistry Macquarie University N.S.W. 2109 Australia; 2 School of Science University of Western Sydney Nepean Kingswood N.S.W. 2747 Australia

## Abstract

A study of the interaction with calf thymus DNA is described of a novel set of chiral ternary complex cations of general form [Ru(N_4_-tet)(phdi)]^2+^ (where N_4_ *-tet* is the chiral linear tetradentate R^*^R^*^-picchxn or R^*^-picchxnMe_2_). Individual equilibrium binding constants (K_B_) have been determined from spectroscopic titrations employing the hypochromism induced in the visible absorbance of the cations on interaction with the nucleic acid. These demonstrate both stereo- and enantioselectivity in the binding interactions. These K_B_ data, together with induced circular dichroism and DNA thermal denaturation results, are all indicative of selective intercalation of the bidentate components of the cations into the nucleobase stack of the duplex. Supportive evidence for a secondary binding mode for the *picchxn* complexes is provided by the different mutagenicity profiles obtained for related cations.


### DNA BINDING OF SOME CHIRAL METALLOINTERCALATORS DERIVED FROM 9,10-PHENANTHRENEDIAMINE

Susan F. Murphy-Poulton[1], Robert S. Vagg *[1], Kymberley A. Vickery # [1] and Peter A. Williams [2]

[1] School of Chemistry, Macquarie University, N.S.W. 2109, Australia
[2] School of Science, University of Western Sydney, Nepean, Kingswood, N.S.W. 2747, Australia

**Abstract**

A study of the interaction with calf thymus DNA is described of a novel set of chiral ternary complex cations of general form $[Ru(N_4\text{-tet})(phdi)]^{2+}$ (where *$N_4$-tet* is the chiral linear tetradentate *R*R*-picchxn* or *R*R*-picchxnMe$_2$*). Individual equilibrium binding constants ($K_B$) have been determined from spectroscopic titrations employing the hypochromism induced in the visible absorbance of the cations on interaction with the nucleic acid. These demonstrate both stereo- and enantioselectivity in the binding interactions. These $K_B$ data, together with induced circular dichroism and DNA thermal denaturation results, are all indicative of selective intercalation of the bidentate components of the cations into the nucleobase stack of the duplex. Supportive evidence for a secondary binding mode for the *picchxn* complexes is provided by the different mutagenicity profiles obtained for related cations.

**Introduction**

The non-covalent binding of metal complex cations to DNA has become a topic of very broad and intense research interest [1-3]. An important determinant of such binding often is the availability of a flat aromatic molecular component in the complex which is capable of intercalating between the stacked bases of the nucleic acid. Several related aromatic bidentate ligands, primarily fused-ring derivatives of 1,10-phenanthroline (*phen*), have been employed commonly for this purpose [4-6]. An alternative bidentate that also has proved to be effective in this regard is 9,10-phenanthrenedione diimine (*phdi*), which has been used as the basis for several intercalating metalloprobes. These generally have been tris-bidentate complex species [3, 7, 8], although some ternary compounds have been studied [9-11].

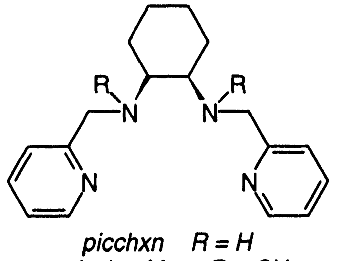

*picchxn R = H*
*picchxnMe$_2$ R = CH$_3$*

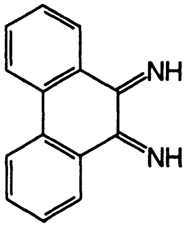

*phdi*

For some time we have been investigating the capabilities of ternary cations of the general form $[Ru(tetradentate)(bidentate)]^{2+}$ to function as stereo- and enantiodiscriminatory intercalating probes of DNA structures [6, 12-14]. In these complexes, the tetradentate is chosen to have characteristics which allow it to govern binding selectivity through helical groove interactions, thereby providing a molecular recognition function. The metal ion provides a substitutionally inert octahedral geometry and the bidentate serves as the intercalating chromophore. Much of the design rationalé for these choices has been outlined in recent reviews [12, 14]. In the work described in this communication, the two tetradentates employed are *N,N'*-di(2-picolyl)-1,2-diaminocyclohexane (*picchxn*), or the corresponding *N,N'*-dimethylated form, *picchxnMe$_2$*, in combination with *phdi* as the bidentate component.

Two different stereoisomeric forms, *cis*-α and *cis*-β, are obtained for this general type of ternary metalloprobe, as depicted in Fig. 1. Each is chiral, providing the opportunity for selectivity in the DNA binding of the different enantiomers. To date the two tetradentates have proved to be enantiospecific in their coordination behaviour, although opposite metal ion chirality results for the two different substitutional forms of the ligand derived from the same hand of the diamine precursor [12, 14]. A further important distinction exists between the two tetradentates. For complexes containing *picchxn,* the *cis*-β geometry allows the possibility of selective secondary intermolecular H-bonding with a host DNA molecule [12].

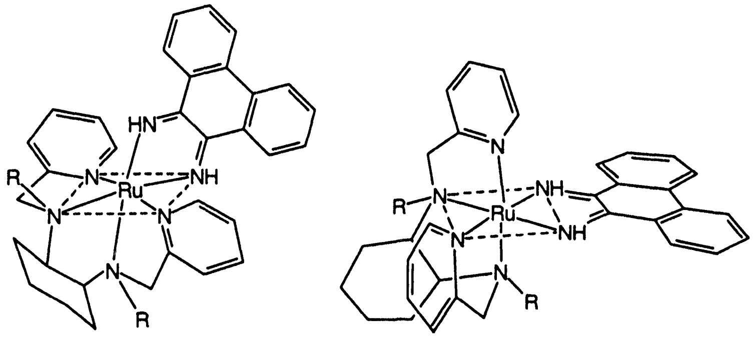

Δ-*cis*-α Λ-*cis*-β

Figure 1. Two stereoisomeric forms of $[Ru(tetradentate)(bidentate)]^{2+}$ metalloprobes

**Materials and Methods**

Ruthenium(III) chloride hydrate, phenanthrene-9,10-diamine and calf thymus DNA were obtained from the Sigma-Aldrich Chemical Company and were used without further purification. The tetradentates *picchxn* and *picchxnMe*$_2$ were synthesised according to published methods [15, 16]. Each *phdi* complex product was obtained *in situ* by the reaction of phenanthrene-9,10-diamine (*phda*) with the appropriate metal complex precursor. Complexes of the *R*R*-picchxn* tetradentate were obtained using the corresponding *cis*-β-[Ru(*R*R**-picchxn)(dmso)Cl]$^+$ isomeric precursors in a stereospecific synthesis [17]. In contrast, both the *cis*-α- and *cis*-β-[Ru(*R*R**-picchxnMe$_2$)(phdi)]$^{2+}$ cations were produced together in a one-pot synthesis [18]. They formed as isomeric mixtures obtained by initial refluxing of equimolar quantities of the *R*R*-picchxnMe*$_2$ tetradentate and $RuCl_3.nH_2O$ in propane-1,2-diol, followed by the addition of *phda* in a two-fold molar excess. The two complex products then were separated by cation-exchange chromatography. In both syntheses, the isomerically pure cations were isolated as either their chloride or hexafluorophosphate salts.

Electronic spectra and thermal DNA denaturation measurements were obtained using a Varian Cary-1 Bio recording spectrophotometer, and circular dichroism (CD) spectra were recorded on a JASCO J-500C spectropolarimeter. Spectroscopic data were acquired in aqueous buffered solutions (10 mM sodium chloride and 10 mM phosphate buffer, pH 7.4) at ambient temperatures. Both CD and NMR methods were used to determine and ensure the diastereoisomeric purity of the complex isomers.

Spectroscopic titrations were carried out to determine the relative intrinsic binding constants, $K_B$, between the individual complexes and calf thymus DNA using the following procedure. Buffered solutions of the nucleic acid were warmed to 90 °C and these then were cooled slowly to ambient temperature to allow gradual renaturing. The annealed solutions were filtered (0.45 μm, Sartorius) and their concentration determined spectrophotometrically using an assumed value for ε of $1.32 \times 10^4$ $mol^{-1}$ L $cm^{-1}$ [19]. Each titration was performed with a constant metalloprobe concentration ([Ru] = $2.5 \times 10^{-5}$ mol $L^{-1}$) and with [DNA] varying, the molar concentration of the nucleic acid being expressed in base-pairs. Absorbance data obtained from the induced hypochromism in the metal ion-*phdi* MLCT regions were used in the determination of binding constants via standard Scatchard plot methods [3, 20]. Circular dichroism (CD) and induced circular dichroism (ICD) plots were obtained using the same spectroscopic titration samples employed for the binding constant determinations. The spectroscopic data were processed using the Microcal Origin® v 4.1 software package.

*Salmonella typhimurium* bacterial strains TA97a and TA98 were obtained from B. N. Ames at the University of California, Berkeley. Mutagenicity (Ames test) assays were carried out following the revised methods as described [21].

### Results

Data from the absorption hypochromism titrations were used to generate Scatchard plots and consequently to determine equilibrium binding constants for the complexes. The results of these calculations are presented in Table 1, together with data for comparable bidentate-based probes binding to calf thymus DNA. As an example, titration data for the Δ-*cis*-β-[Ru(*SS*-picchxn)(phdi)]$^{2+}$ cation are shown plotted in Fig. 2. There it may be seen that a maximum of *ca.* 25% absorption hypochromism is reached at a DNA:probe ratio of *ca.* 3.6 and a corresponding maximum change in the circular dichroism spectrum of the system also is observed at this ratio. The use of hypochromism data for equilibrium binding constant calculation also is exemplified for this cation in the Scatchard plot shown in Fig 2.

Table 1: Equilibrium Binding Constants ($K_B$ x $10^{-4}$ M $bpr^{-1}$) for Metalloprobes with Calf Thymus DNA

| Complex | $K_B$ (x $10^{-4}$) | Ref |
|---|---|---|
| Δ-*cis*-α-[Ru(*RR*-picchxnMe$_2$)(phdi)]$^{2+}$ | 12.0(13) | this work |
| Δ-*cis*-β-[Ru(*RR*-picchxnMe$_2$)(phdi)]$^{2}$ | 2.3(2) | this work |
| Λ-*cis*-α-[Ru(*SS*-picchxnMe$_2$)(phdi)]$^{2+}$ | 8.1(11) | this work |
| Λ-*cis*-β-[Ru(*SS*-picchxnMe$_2$)(phdi)]$^{2+}$ | 29(5) | this work |
| Δ-*cis*-α-[Ru(*RR*-picchxnMe$_2$)(dpq)]$^{2+}$ | 1.23(6) | 14 |
| Δ-*cis*-β-[Ru(*RR*-picchxnMe$_2$)(dpq)]$^{2}$ | 1.62(7) | 14 |
| Λ-*cis*-α-[Ru(*SS*-picchxnMe$_2$)(dpq)]$^{2+}$ | 4.90(8) | 14 |
| Λ-*cis*-β-[Ru(*SS*-picchxnMe$_2$)(dpq)]$^{2+}$ | 1.10(10) | 14 |
| Λ-*cis*-α-[Ru(*SS*-picchxnMe$_2$)(phen)]$^{2+}$ | 0.25(5) | 14 |
| Δ-*cis*-β-[Ru(*SS*-picchxn)(phdi)]$^{2+}$ | 7.1(5) | this work |
| *rac*-β-[Ru(picchxn)(phdi)]$^{2+}$ | 7.1(3) | this work |
| *rac*-[Ru(phen)$_3$]$^{2+}$ | 0.55(10) | 7 |
| *rac*-[Ru(bpy)$_2$(phdi)]$^{2+}$ | 4.8(10) | 7 |
| *rac*-[Ru(phen)$_2$(phdi)]$^{2+}$ | 4.7(10) | 7 |
| Δ-[Ru(phen)$_2$(phdi)]$^{2+}$ | 6.2 | 3 |
| Λ-[Ru(phen)$_2$(phdi)]$^{2+}$ | 2.6 | 3 |
| Δ-[Ru(bpy)$_2$(phdi)]$^{2+}$ | 2.6 | 3 |
| Λ-[Ru(bpy)$_2$(phdi)]$^{2+}$ | 2.2 | 3 |

As an example, the significant circular dichroism induced in the two Δ-*cis*-β probes' MLCT chromophores in the 500-550 nm region upon addition of calf thymus DNA is demonstrated in Fig. 3. In each case, a maximum degree of induction is achieved at the DNA:Ru ratio of *ca.* 3.6. The similarities in these CD spectral features are also of interest, particularly considering that the tetradentate ligand components are of opposite chirality. The results of thermal denaturation studies on calf thymus DNA in the presence of varying amounts of Δ-*cis*-β-[Ru(*SS*-picchxn)(phdi)]$^{2+}$ are given in Table 2.

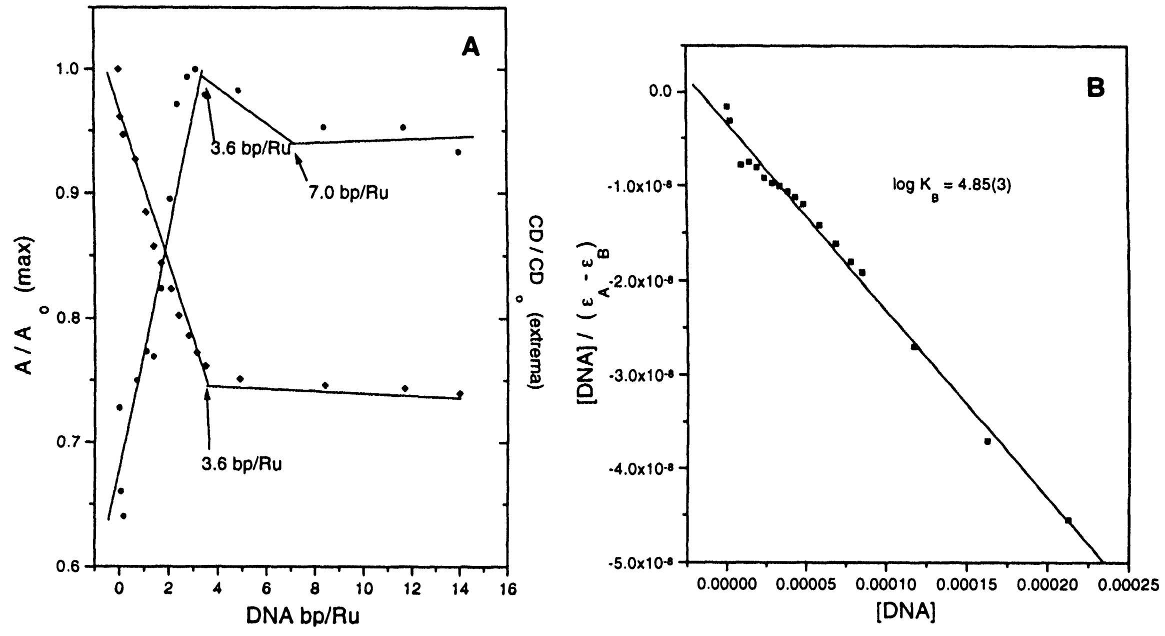

Figure 2. (A) Changes in relative absorbance and circular dichroism with increasing DNA:Ru ratio for the interaction of Δ-*cis*-β-[Ru(*SS*-picchxn)(phdi)]$^{2+}$ with calf thymus DNA. (B) Scatchard plot derived from relative absorbance data shown in (A).

Table 2. Thermal melting data for calf thymus DNA in the presence of Δ-*cis*-β-[Ru(*SS*-picchxn)(phdi)]$^{2+}$ at different Ru:DNA ratios.

| DNA:Ru ratio | Tm (°C) | ΔTm (C°) |
|---|---|---|
| DNA alone | 61.0 | 0.0 |
| 10.2 | 67.5 | 6.5 |
| 7.0 | 89.5 | 28.5 |
| 3.6 | 87.2 | 26.2 |

### Discussion

The high equilibrium binding constant values of *ca.* $10^5$ calculated for the *phdi*-based ternary complexes (Table 1) indicate strong binding affinities which are significantly larger than for *phen*-based probes and which generally are larger than their *dpq* analogues. The range of values obtained for the four different diastereomeric forms of the *picchxnMe*$_2$ cations indicate that these probes are stereodiscriminatory in binding DNA. In particular, for the pair of *cis*-β complexes, a ten-fold enantiomeric preference is indicated in favour of the Λ hand. This contrasts with the minor enantiodiscriminations observed for the tris-bidentate probe systems incorporating the *phdi* chromophore.

Further, a comparison of the $K_B$ values for the Δ-*cis*-β isomeric forms of the two different tetradentates indicates a significant binding preference for the unsubstituted *picchxn* derivative. This may result from its lower steric bulk, which would allow a closer fit of the cation into the duplex, or it may be due to H-bonding involving the proximal amine group of the cation with nucleobase acceptor groups on the nucleic acid molecules [12]. The molecular structure of this cation (Fig 4), derived from an X-ray analysis, both confirms the absolute configuration of each chiral centre and shows the positioning of the amine proton below the *phdi* intercalating fragment. It also demonstrates the presence of significant torsional distortion in this *phdi* bidentate component. A similar distortion from planarity has been observed in the structure of a related tris-bidentate probe, which has been suggested as a possible source of enantiodiscrimination in DNA binding of *phdi*-based metallointercalators [22]. However, the values of the binding constants reported for the tris-bidentate probes (Table 1) would not support this proposal.

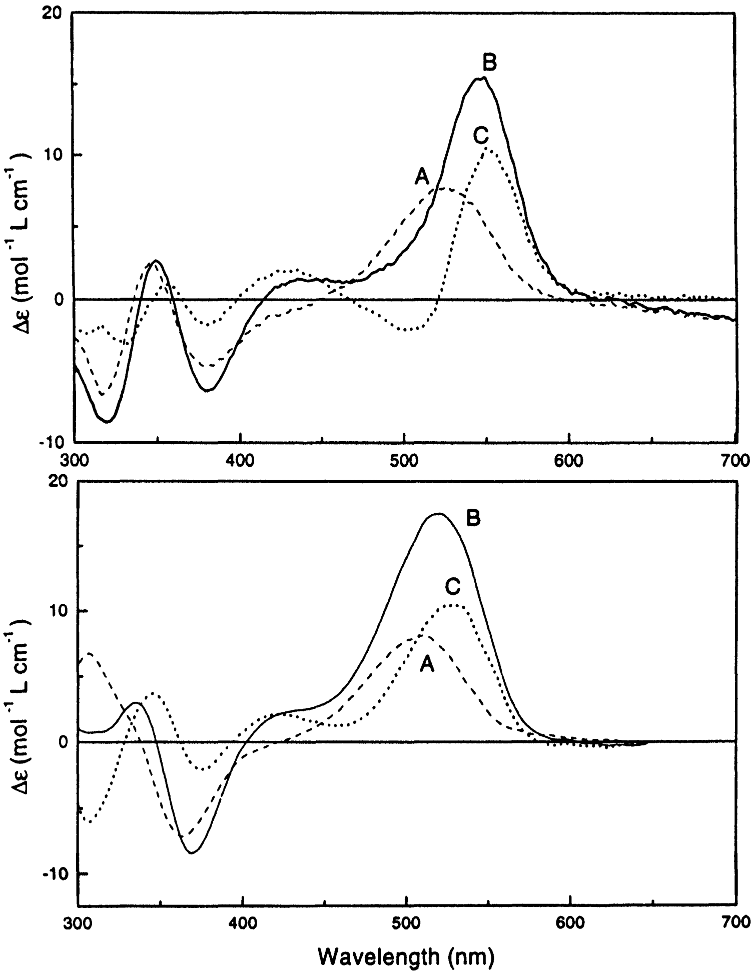

Figure 3. Circular dichroism (CD) spectra recorded in the visible range for the Δ-*cis*-β-[Ru(*SS*-picchxn)(phdi)]$^{2+}$ (top) and Δ-*cis*-β-[Ru(*RR*-picchxnMe$_2$)(phdi)]$^{2+}$ (bottom) cations in the absence (A) and presence (B) of calf thymus DNA. The induced circular dichroism (ICD) resulting from the probe-nucleic acid interaction is shown as (C) in each case.

Another interesting distinction between the two *picchxn*- and *picchxnMe$_2$*-based cations lies in their observed fluorescence properties. Preliminary results show that the non-methylated *picchxn* derivative alone demonstrates significantly enhanced fluorescence upon interaction with calf thymus DNA, whereas the *picchxnMe$_2$* complex shows no evidence of fluorescence activity.

The effect of the Δ-*cis*-β-[Ru(*SS*-picchxn)(phdi)]$^{2+}$ cation on the thermal denaturing of calf thymus DNA is quite pronounced (Table 2). The large increase in Tm observed at lower DNA-probe ratios is consistent with an intercalative binding mode for the cation, and hence for *phdi*-based probes in general. The significant hypochromism and ICD observed also indicate this type of binding. The common observation of maximum spectroscopic effects occurring at a DNA:Ru ratio of 3.6 indicates this ratio to be the limiting stoichiometry for the DNA-binding interaction. This would represent the point at which saturation occurs of the available intercalation sites on the nucleic acid by the probe molecules, and would be the limit for DNA-probe adducts derived from the *cis*-β tetradentate geometry.

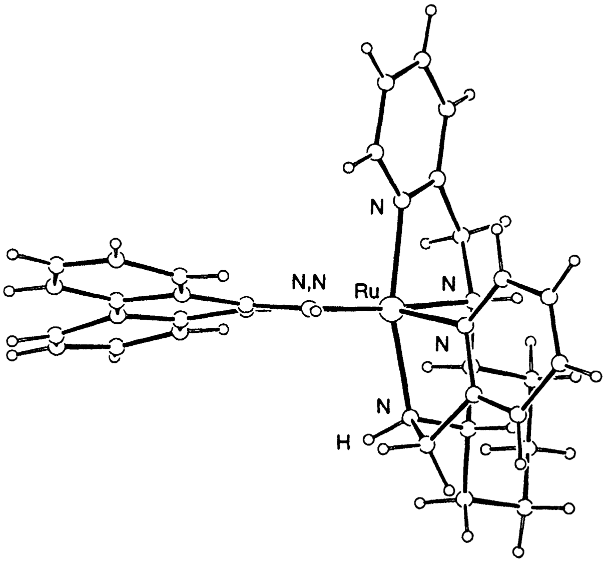

Figure 4. A view of the Δ-*cis*-β-[Ru(*SS*-picchxn)(phdi)]$^{2+}$ cation projected along the N..N direction of the *phdi* bidentate ligand (T.W. Hambley *et al.*, unpublished results)

Further support for a secondary binding mode arises from the bacterial mutagenicity (Ames test) activity spectrum determined for the various isomeric forms of the *phdi* cations, where markedly different results were found. Each *S. typhimurium* tester strain detects different mutational events as a result of DNA interactions with the test compound [23]. Testing of Λ-*cis*-β-[Ru(*SS*-picchxn)(phdi)]$^{2+}$ showed high mutagenicity in both strains TA98 and TA97a, in contrast to the positive response observed in TA97a (only) for the Δ-*cis*-β-[Ru(*RR*- and Λ-*cis*-β-[Ru(*SS*-picchxnMe$_2$)(phdi)]$^{2+}$ analogues [18]. These results suggest an additional binding mode for this complex, which is not available to the methylated analogue and is proposed to be attributable to its H-bonding potential.

### Acknowledgments

The funding support of the Australian Research Council and the Swedish Institute is gratefully acknowledged.

### References

1. C.M. Dupeureur and J.K. Barton, *Inorg. Chem.*, (1997), **36**, 33.
2. B. Nordén, P Lincoln, B. ≈kerman and E. Tuite, *Metal Ions in Biological Systems: Probing of Nucleic Acids by Metal Ion Complexes of Small Molecules*, (1996), **33**, Marcel Dekker, Inc.
3. K. Naing, M. Takhashi, M. Taniguchi, and Y. Yamagishi, *Inorg. Chem.*, (1995), **34**, 350.
4. J.K. Barton, J.J. Dannenberg and. A.L. Raphael, *J. Am. Chem. Soc.*, (1982), **104,** 4967.
5. S-D. Choi, M-S. Kim, S.K. Kim, P. Lincoln, E. Tuite and B. Nordén, *Biochem.*, (1997), **36,** 214.
6. J.R. Aldrich-Wright, I. Greguric, R.S. Vagg, K.A. Vickery and P.A. Williams, *J. Chromatog. A.*, (1995), **718**, 436.
7. A.M. Pyle, J.P. Rehmann, R. Meshoyrer, C.V. Kumar, N.J. Turro, and J.K. Barton, *J. Am. Chem. Soc.*, (1989), **111**, 3051.
8. A. Sitlani and J.K. Barton, *Biochem.*, (1994), **33**, 12100.

9. A.H. Krotz, B.P. Hudson and J.K. Barton, *J. Am. Chem. Soc.,* (1993), **115**, 12577.
10. A.H. Krotz, L.Y. Kuo, and J.K. Barton, *Inorg. Chem.*, (1993), **32**, 5963.
11. A.H. Krotz and J.K. Barton, *Inorg. Chem.*, (1994), **33**, 1940.
12. J.R. Aldrich-Wright, R.S. Vagg and P.A. Williams, *Coord. Chem. Rev.,* (1997), **166**, 361.
13. E.M. Proudfoot, J.P. Mackay, R.S. Vagg, K.A. Vickery, P.A. Williams and P.H. Karuso, *J. Chem. Commun.,* (1997), 1623.
14. K.A. Vickery, A.M. Bonin, P.A. Williams and R.S. Vagg, in *Structure, Motion, Interaction and Expression of Biological Macromolecules,* H.S. Sarma and M.H. Sarma (Editors), Adenine Press, New York, U.S.A. (1998), Volume 1, pp 195-206.
15. T.J. Goodwin, R.S. Vagg and P.A. Williams, *J. Proc. Royal Soc. N. S. W.,* (1984), **117**, 1.
16. R.R. Fenton, F.S. Stephens, R.S. Vagg and P.A. Williams, *Inorg. Chim. Acta,* (1991), **182**, 67.
17. S.M. Murphy-Poulton, *MSc Thesis,* Macquarie University, Australia, (1998).
18. K.A. Vickery, *PhD Thesis,* Macquarie University, Australia, (1998).
19. R.B. Inman and R.L. Baldwin, *J.Mol. Biol.,* (1962), **5**, 172.
20. D.E.V. Schmechel and D.M. Crothers, *Biopolymers*, (1971), **10**, 465.
21. D.M. Maron and B.N. Ames, *Mutat. Res.,* (1983), **113**, 173.
22. Johann, T. W., Barton, J. K., *Phil. Trans. R. Soc. Lond. A* , 354, 299 (1996).
23. It is known that the frameshifting mutagenic properties of acridines and other DNA intercalators give a characteristic pattern of responses, showing that acridines (at least) which intercalate cause frameshift mutations which can be detected in *S. typhimurium* TA1537 and TA97a strains, rather than in TA98. Mutagenesis in TA98 appears to be associated with compounds which can cause DNA covalent adducts [E.C McCoy, E.J. Rosenkranz, L.A. Petrullo and H.S. Rosenkranz, *Mutat. Res.,* (1981), **90**, 21; D.M. Maron and B.N. Ames, *Mutat. Res.,* (1983), **113,** 173; L.R. Ferguson, W.A. Denny, D.G. MacPhee, (1985), *Mutat. Res,* **157**, 29; L.R. Ferguson and W.A. Denny, (1990), *Mutag.,* **5(6)**, 529.]

**Received: July 1, 1998 - Accepted: July 14, 1998**

